# Genotypic and phenotypic profiles of *EYS* gene-related retinitis pigmentosa: a retrospective study

**DOI:** 10.1038/s41598-022-26017-0

**Published:** 2022-12-13

**Authors:** Ragkit Suvannaboon, Aulia Rahmi Pawestri, Worapoj Jinda, Aekkachai Tuekprakhon, Adisak Trinavarat, La-ongsri Atchaneeyasakul

**Affiliations:** 1 Department of Ophthalmology, Faculty of Medicine Siriraj Hospital, Mahidol University, 2 Wanglang Road, Bangkok Noi, Bangkok, 10700 Thailand; 2 Faculty of Medicine, Universitas Brawijaya, Malang, Indonesia; 3 Research Division, National Cancer Institute, Bangkok, Thailand

**Keywords:** Genetics, Diseases, Molecular medicine, Pathogenesis

## Abstract

Retinitis pigmentosa (RP) affects 1:5000 individuals worldwide. Interestingly, variations in 271 RP-related genes are indicated to vary among populations. We aimed to evaluate the genetic prevalence and phenotypic profiles of Thai patients with RP. The clinical and whole exome sequencing data of 125 patients suggestive of inherited retinal diseases (IRD), particularly non-syndromic RP, were assessed. We found a total of 258 variants (63% of which remained unavailable in the ClinVar database) in 91 IRD-associated genes. Among the detected genes, the eyes shut homolog (*EYS*) gene showed the highest prevalence. We also provide insights into the genotypic, baseline, and follow-up clinical presentations of seven patients with disease-causing *EYS* variations. This study could provide comprehension of the prevalence of RP-related genes involved in the Asian population. It might also provide information to establish advanced and personalised therapy for RP in the Thai population.

## Introduction

Retinitis pigmentosa (RP, OMIM #268000) is an umbrella term describing a group of progressive inherited retinal diseases (IRD), primarily affecting photoreceptors or retinal pigment epithelium (RPE), impacting approximately one in 5000 individuals worldwide^1,2^. To date, at least 271 genes have been identified to be related to RP^3^. As such, the inheritance patterns of RP vary widely, including autosomal dominant (adRP), autosomal recessive (arRP), and X-linked (xlRP).

Genetic mutations result from several plausible mechanisms, most of them involving biochemical pathways, including apoptosis, light damage, ciliary transport dysfunction, and endoplasmic reticulum stress, leading to the dysregulation or dysfunction of photoreceptors, commonly rod cells^4^. Considering that rod cells mainly function in low-light vision, the loss of rod cells often result in poor night vision, night blindness (nyctalopia), and gradual peripheral vision loss^5^. The apoptosis, one of the most essential pathways in the pathophysiology of cell loss, could spread via the cell-to-cell communication pathway. Therefore, large destruction of rod cells could induce the apoptosis of other retinal structures, including RPE and cone cells. This, in turn, results in abnormal colour perception, colour blindness, and other symptom spectrum in RP^5^.

The eyes shut homolog (*EYS*) gene mutations are one of the common causes of the autosomal recessive RP, especially in the Asian population^6^. The exact function of the EYS protein is still uncertain, but it has been described to be involved in maintaining the integrity of photoreceptor cells^7^. Patients with *EYS* mutations present with a typical RP phenotype, but there are also reports of cone-rod dystrophy caused by *EYS* mutations^8^. In the Portuguese and Brazilian population, mutations in the *EYS* gene have been suggested to be one of the major causes of sector RP, an unusual form in which the clinical signs present only in one or two fundus quadrants, beside the Rhodopsin (*RHO*) gene mutations^9^.

Considered as one of the biggest expressed genes expanding for 2 Mb of genomic DNA, the *EYS* is approximately 10.5 Kb with 43 exons, giving rise to a multi-domain protein of 3145-3156 amino acids (aa). This protein consists of 21 epidermal growth factor (EGF)-like and 5 laminin G domains at the N- and C-terminus, respectively, with additional interspace of EGF-like domains^10,11^. Four isoforms have been identified. Isoform 1 (Uniprot I.D Q5T1H1-1, 3144 aa) and 4 (Q5T1H1-3, 3165 aa) are among the longest isoforms found in the retina. Despite being similar to isoform 1, isoform 4 contains additional amino acids at the C-terminal domain, between the 41st and 42nd exon. On the other hand, the shorter isoforms, 2 (Q5T1H1-4, 619 aa) and 3 (Q5T1H1-3, 594 aa), lack the 16 EGF-like domains with varying lengths in the C-terminal domain^12^. Studies from the *Eys* knock-out zebra fish models revealed their function in maintaining the photoreceptor structural integrity and architecture of the Eys^13^.

We previously reported a pilot study identifying the variant-associated IRDs, including RP, Leber congenital amaurosis (LCA), and cone-rod dystrophy (CRD), in 86 genes. Seventeen variants were detected in 11 out of 20 patients; the detected variants were located in 5 autosomal recessive genes (*CRB1, C8orf37, EYS, PROM1*, and *USH2A*) and one X-linked recessive gene (*RP2*)^14^.

In this study, we reported the prevalence of the RP-associated gene variations in 125 Thai patients. The potential RP-causing variants were explored with a particular highlight for clinical insights related to disease-causing *EYS* variations. Since the prevalence of IRD-related disease-causative genes varied among populations, together with our previously reported data, this could provide a more powerful information on the prevalence of RP-related genes involved in the Asian population. In the future, it might also contribute basic information for the initiation of advanced and personalised therapy for RP in the Thai population.

## Materials and methods

### Ethical statements

All procedures were performed in accordance with the relevant guidelines and regulations and had been approved by the institutional review board of the Faculty of Medicine Siriraj Hospital, Mahidol University (ethical approval number Si.092/2022, dated February 2, 2022). Informed consent related to data usage and publishing the information/image(s) in an online open access publication was obtained from all participants.

### Study design

This retrospective study utilised clinical and whole exome sequencing (WES) data of Thai patients with symptoms and clinical signs suggestive of IRD, particularly non-syndromic RP, attending the out-patient Ophthalmology Clinic in Siriraj Hospital. The clinical diagnosis of RP was made by experienced ophthalmologist. Demographic data, subjective complaints, and family history was recorded. The supporting clinical data at baseline and last follow up comprised clinical presentations, family history, best corrected visual acuity (BCVA) with a grading as reported in our previous study^15^, visual field (VF), electroretinography (ERG), optical coherence tomography (OCT), colour vision, and fundus photography.

### Identification of RP-related gene mutation and variant detection

The data of WES and variant identification procedures were performed and described previously by Jinda et al.^14^. All identified variants in the retinal-associated genes in the RetNet database (accessed March 9th, 2016) were used to identify the causative variants. Other interesting deleterious variants which might potentially impair the function of the IRD-associated genes were also collected.

To further identify the disease-associated variants, we classified the clinical significance based on ClinVar. However, reported variants with conflicting interpretations among studies or those unavailable in the ClinVar database were classified based on the American College of Medical Genetics (ACMG) guideline^16^. The classification ranged from benign, likely benign, variant of uncertain significance (VUS), likely pathogenic, and pathogenic. Considering both likely pathogenic and pathogenic to be the cause of the disease, variants in both classifications were used to define the causative genes. Additionally, an X-linked RP gene (in male patients) and an autosomal dominant RP gene containing at least one pathogenic/likely pathogenic variant was suspected to be a disease-causative gene. An autosomal recessive RP gene containing at least one pathogenic variant in the homozygous or compound heterozygous state was also suspected to be a disease-causative gene.

## Results

### Prevalence of IRD genes in Thai patients

**Figure 1:**
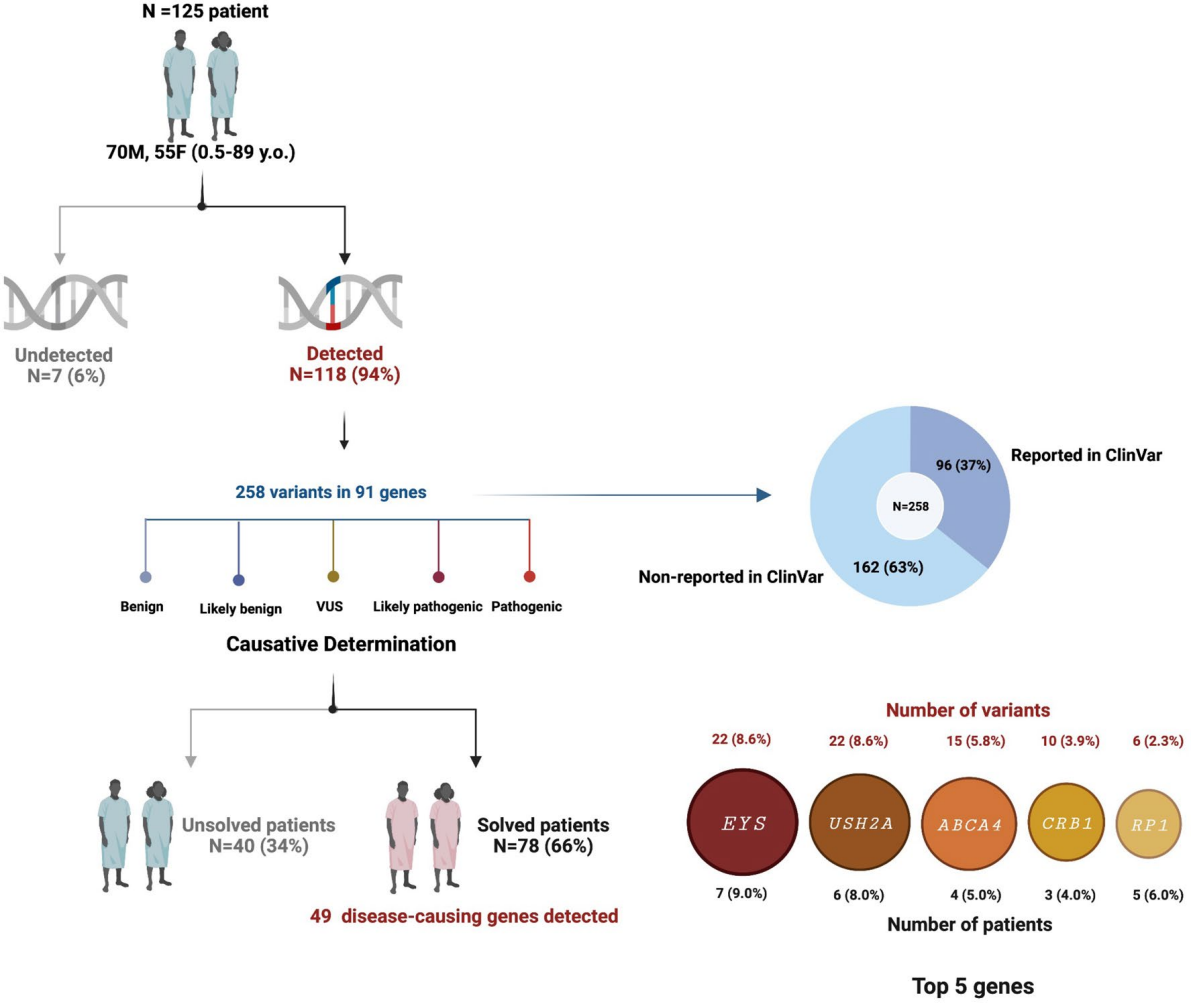
WES data of 125 Thai patients with IRD. Variants were detected in 94% of the patients. Based on variant classification, 49 genes were determined to cause diseases in 66% of the patients, with the *EYS, USH2A, ABCA4, CRB1*, and *RP1* showing the highest prevalence. This figure was created using Biorender.

In 2014, we reported a pioneer batch of the WES data in 20 unrelated Thai patients with IRDs^14^. Here, we provided more information on the prevalence of IRD-related genes and their variants in 125 unrelated Thai patients. With the overall average read depth of approximately 150X, we identified variations in 91 IRD-associated genes in 94.40% (118/125) of patients (Fig. 1 and Supplementary Table S1). Within 91 identified genes, a total of 258 variants were detected. The highest number of variants was found in *EYS* (8.6%, 22/258) and *USH2A* (8.6%, 22/258), followed by *ABCA4* (5.8%, 15/258), *CRB1* (3.9%, 10/258), and *RP1* (2.3%, 6/258) (Fig. 1). The list of other genes accounting for less than 2% is shown in Supplementary Table S1. Interestingly, 162 variants found in this study have not been reported in ClinVar (as of February 25th, 2022) (Fig. 1 and Supplementary Table S1). Consequently, 49/91 genes were determined to be the cause of disease in 78 out of 118 Thai patients (62% detection rate). The top 5 prevalent genes accounted for 32% included *EYS* (9%, 7/78 patients), *USH2A* (8%, 6/78), *ABCA4* (5%, 4/78), *CRB1* (4%, 3/78), and *RP1* (6%, 5/78) (Fig. 1 and Supplementary Table S1).

### Variants in *EYS* gene

**Figure 2:**
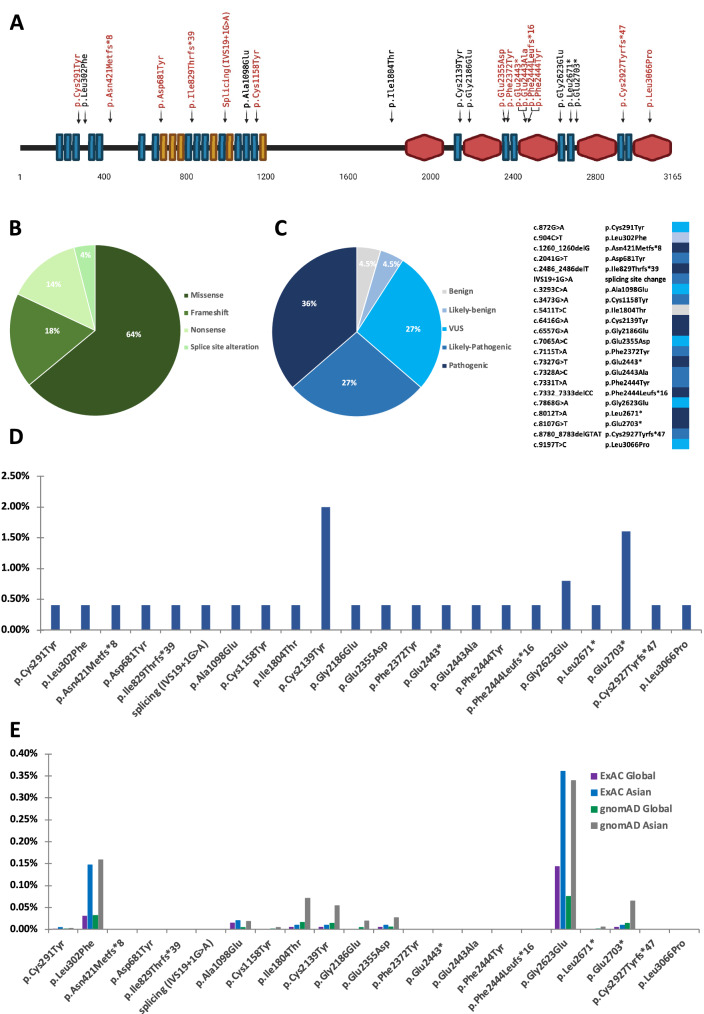
Variants in the *EYS* gene. (**A**) Schematic diagram of the human EYS protein (isoform 4, accession: NM_001292009.2), with 21 EGF-like (blue rectangles), 6 EGF-like calcium-binding (red rectangles), and 5 Laminin-G domains (red hexagons). Novel and reported variants are depicted in red and black, respectively. Types (**B**) and classification (**C**) of the detected variants (N = 22). Allele frequency of the detected variants in this cohort (**D**) and in the general population from ExAC and gnomAD (**E**).

Variations in the *EYS* gene had the highest prevalence in our Thai patient population. As shown in Fig. 2A, the human EYS protein consists of 3165 aa (based on isoform 4, accession: NM_001292009.2, uniprot: Q5T1H1-3). The location of all 22 variants (14 non-reported and 8 reported) identified in our study were mapped to the EYS protein diagram. Most of the variants resided within either the EGF-like cluster at the N-terminus side or the laminin G-like cluster at C-terminus side. Fifty-five percent (12/22) of the variants were located in multiple EGF-like domains, 27% (6/22) were found in the laminin G-like domain 2, 3, and 5. The remaining 18% (4/22) of the variants were not located in the domain regions of the EYS protein (Fig. 2A and Table 1), where these were reported as pathogenic if manifested as nonsense and frameshift variants. There were 64% (14/22) of missense mutation, 18% (4/22) frameshift, 14% (3/22) nonsense, and 4% (1/22) splice site alteration (Fig. 2B and Table 1). Among these 22 variants, pathogenic variants accounted for 36% (8/22), followed by likely pathogenic (27%, 6/22), VUS (27%, 6/22), likely benign (4.5%, 1/22), and benign (4.5%, 1/22) (Fig. 2C and Table 1). It is worth to note that all nonsense variants found (p.Glu2443* (Laminin G-like 3), p.Leu2671* (EGF-like 25), and p.Glu2703*) were pathogenic variants. Frameshift variants were either pathogenic or likely-pathogenic. Most of the missense mutations classified as VUS were scattered in the EGF-like domains (domain 3, 18, 22, and 24), except for one which was in the Laminin G-like domain 5. One benign (p.Ile1804Thr) and likely benign (p.Leu302Phe) were missense variants located neither in EGF-like nor Laminin G-like domains.

**Table 1:** Variants of the *EYS* gene.

No	Variant	Amino acid change	Variant type	Location in EYS protein	Variant classification	Allele frequency (%)					Reference
						ExAC		gnomAD—exomes		This cohort	
						Global	Asian	Global	Asian		
1	c.872G>A	p.Cys291Tyr	Missense	EGF-like 3	VUS	0.0008%	0.004%	0.0004%	0.002%	0.4% (1/250)	rs749086805
2	c.904C>T	p.Leu302Phe	Missense	–	Likely benign​	0.0305%	0.147%	0.0315%	0.159%	0.4% (1/250)	VCV000760888.3
3	c.1260_1260delG	p.Asn421Metfs*8	Frameshift	–	Pathogenic	N/A	N/A	N/A	N/A	0.4% (1/250)	Jinda, 2014
4	c.2041G>T	p.Asp681Tyr	Missense	EGF-like 8	Likely pathogenic	N/A	N/A	N/A	N/A	0.4% (1/250)	Novel
5	c.2486_2486delT	p.Ile829Thrfs*39	Frameshift	EGF-like 11	Pathogenic	N/A	N/A	N/A	N/A	0.4% (1/250)	Novel
6	IVS19+1G>A	splicing site change	Splicing	EGF-like 15	Likely pathogenic	N/A	N/A	N/A	N/A	0.4% (1/250)	Novel
7	c.3293C>A	p.Ala1098Glu	Missense	EGF-like 18	VUS	0.014%	0.02%	0.0045%	0.018%	0.4% (1/250)	VCV000990050.1
8	c.3473G>A	p.Cys1158Tyr	Missense	EGF-like 19	VUS	N/A	N/A	0.0007%	0.004%	0.4% (1/250)	rs1346175770
9	c.5411T>C	p.Ile1804Thr	Missense	–	Benign​​	0.005%	0.01%	0.0157%	0.071%	0.4% (1/250)	VCV000797211.4
10	c.6416G>A	p.Cys2139Tyr	Missense	EGF-like 21	Pathogenic	0.005%	0.01%	0.0144%	0.054%	2% (5/250)	VCV000189230.17
11	c.6557G>A	p.Gly2186Glu	Missense	Laminin G-like 2	Pathogenic	N/A	N/A	0.004%	0.019%	0.4% (1/250)	VCV000143108.4
12	c.7065A>C	p.Glu2355Asp	Missense	EGF-like 22	VUS	0.005%	0.01%	0.0058%	0.027%	0.4% (1/250)	rs749617474
13	c.7115T>A	p.Phe2372Tyr	Missense	EGF-like 23	Likely pathogenic	N/A	N/A	N/A	N/A	0.4% (1/250)	rs1004559050
14	c.7327G>T	p.Glu2443*	Nonsense	Laminin G-like 3	Pathogenic	N/A	N/A	N/A	N/A	0.4% (1/250)	rs1459422877
15	c.7328A>C	p.Glu2443Ala	Missense	Laminin G-like 3	Likely pathogenic	N/A	N/A	N/A	N/A	0.4% (1/250)	Novel
16	c.7331T>A	p.Phe2444Tyr	Missense	Laminin G-like 3	Likely pathogenic	N/A	N/A	N/A	N/A	0.4% (1/250)	Novel
17	c.7332_7333delCC	p.Phe2444Leufs*16	Frameshift	Laminin G-like 3	Pathogenic	N/A	N/A	N/A	N/A	0.4% (1/250)	Novel
18	c.7868G>A	p.Gly2623Glu	Missense	EGF-like 24	VUS	0.143%	0.36%	0.0748%	0.339%	0.8% (2/250)	VCV000556919.7
19	c.8012T>A	p.Leu2671*	Nonsense	EGF-like 25	Pathogenic​	N/A	N/A	0.0013%	0.006%	0.4% (1/250)	VCV000143113.3
20	c.8107G>T	p.Glu2703*	Nonsense	–	Pathogenic​	0.005%	0.01%	0.0143%	0.065%	1.6% (4/250)	VCV000853127.6
21	c.8780_8783delGTAT	p.Cys2927Tyrfs*47	Frameshift	EGF-like 26	Likely pathogenic	N/A	N/A	N/A	N/A	0.4% (1/250)	Novel
22	c.9197T>C	p.Leu3066Pro	Missense	Laminin G-like 5	VUS	N/A	N/A	N/A	N/A	0.4% (1/250)	Novel

EGF-like domain, epidermal growth factor-like domain; VUS, variant of unknown significance; ExAC, Exome Aggregation Consortium; gnomAD, Genome Aggregation Database.

The allele frequency (AF) was further determined to evaluate the disease-associated variants in this cohort (125 patients, 250 alleles). The AF in this study and in the general population from the Exome Aggregation Consortium (ExAC) and the Genome Aggregation Database (gnomAD) were presented in Table 2 and Fig. 2D and E. The percentage of AF of 22 variants in the *EYS* gene ranged from 0.4 to 2%. The pathogenic variant p.Cys2139Tyr (2%, 5/250), found in EGF-like domain 21, showed the highest percentage, followed by p.Glu2703* (1.6%, 4/250) and p.Gly2623Glu (0.8%, 2/250). As a comparison, data of 8 variants (p.Leu302Phe, p.Ala1098Glu, p.Ile1804Thr, p.Cys2139Tyr, p.Gly2186Glu, p.Glu2355Asp, p.Gly2623Glu, p.Glu2703*) were retrieved from ExAC and gnomAD, where both Asian and global population showed an AF of less than 0.4% (Table 1). It is interesting to note that the AF of 8 variants in the Asian population were notably higher than the global population (Fig. 2E).

**Table 2:** Baseline demographic clinical presentation.

Patient ID	Gender	Age of onset (years)	Age at diagnosis (years)	Family history of RP	Presenting symptoms	Eye	BCVA (logMAR)	Visual field (degree)				Colour vision	ERG
								S	N	I	T		
P1	F	19	29	No	Reduced night vision	OD	0.18	12	12	12	12	Normal	Non-recordable
						OS	0.18	12	12	12	12		
P2	F	47	49	No	Reduced night vision, blurred vision, impaired colour vision	OD	0.6	10	10	10	10	Total colour blindness	Non-recordable
						OS	0.48	10	12	10	10		
P3	M	49	50	Possible (father with reduced night vision, paternal uncle with bad vision)	Reduced night vision	OD	0.78	37	10	70	45	Total colour blindness	Reduced flicker, non-recordable scotopic
						OS	0.6	40	10	70	45		
P4	F	unknown	42	No	Narrow visual field	OD	0.6	NE	NE	NE	NE	Total colour blindness	Non-recordable
						OS	0.48	NE	NE	NE	NE		
P5	M	48	50	No	Reduced night vision, blurred vision, impaired stereopsis	OD	0.2	18	18	20	30	Normal	Non-recordable
						OS	0.2	16	18	20	25		
P6	F	15	46	No	Reduced night vision, blurred vision, narrow visual field	OD	2.3	NE	NE	NE	NE	NE	Non-recordable
						OS	2.7	NE	NE	NE	NE		
P7	F	45	46	Possible (elder brother and elder sister with blurred vision)	Blurred vision	OD	0.6	NE	NE	NE	NE	Abnormal colour vision	Reduced flicker, reduced scotopic
						OS	0.6	NE	NE	NE	NE		

BCVA, best corrected visual acuity; ERG, electroretinography; F, female; M, male; logMAR, logarithm of the minimum angle of resolution; OD, oculi dextra; OS, oculi sinistra; RP, retinitis pigmentosa; S, superior; N, nasal; I, inferior; T, temporal; NE, not evaluated.

### Clinical presentation of patients with disease-causing *EYS* variants

A total of 125 Thai patients with signs of visual impairment suspected of IRD were recruited in this study, consisting of 70 males and 55 females, with a median age at diagnosis of 40 years (ranging from 0.5 to 89 years) (Fig. 1). We found *EYS* variants in 18/125 patients. Applying the criteria of disease-associated variant identification (see method), only 7/18 patients (assigned P1-7) were conclusively identified to have the *EYS* as the causative gene for RP, while patient P8-18 have no disease-causing variants in the *EYS* gene (Supplementary Table S2).

In these 7 patients, which consisted of five females and two males, the median age of onset and age at diagnosis was 37 years (15–49 years) and 44.5 years (29–50 years), respectively (Fig. 1, Table 2). From history taking, the most common initial symptoms were reduced night vision occurring in five out of seven patients, followed by blurred vision 57.1% (4/7), and narrow visual field 28.6% (2/7). Other presenting symptoms include impaired colour vision, impaired perception of depth/stereopsis, and distorted vision, each of which occurring in 14.3% (1/7) of patients. Two patients had family members with visual impairments, while the rest had no known RP-related family history (Table 2).

The ophthalmological examinations revealed that 28.6% (2/7) presented with normal vision or mild visual loss (BCVA in both eyes at LogMAR < 0.54); 57.1% (4/7) had moderate visual loss (both eyes at LogMAR 0.54—1.00); and 14.3% (1/7) was considered as legal blindness (LogMAR ≥ 1.00). The VF data were obtained from only 4 patients, all of whom, showing narrowed VF. Total colour blindness was observed in 42.9% (3/7), abnormal colour vision in 14.3% (1/7), normal colour vision in 28.6% (2/7), and unable to be evaluated in 14.3% (1/7) of patients due to low vision. All patients except one had a non-recordable ERG (Table 2).

### Phenotypic and genotypic presentations of unrelated patients with *EYS*-associated RP

**Figure 3:**
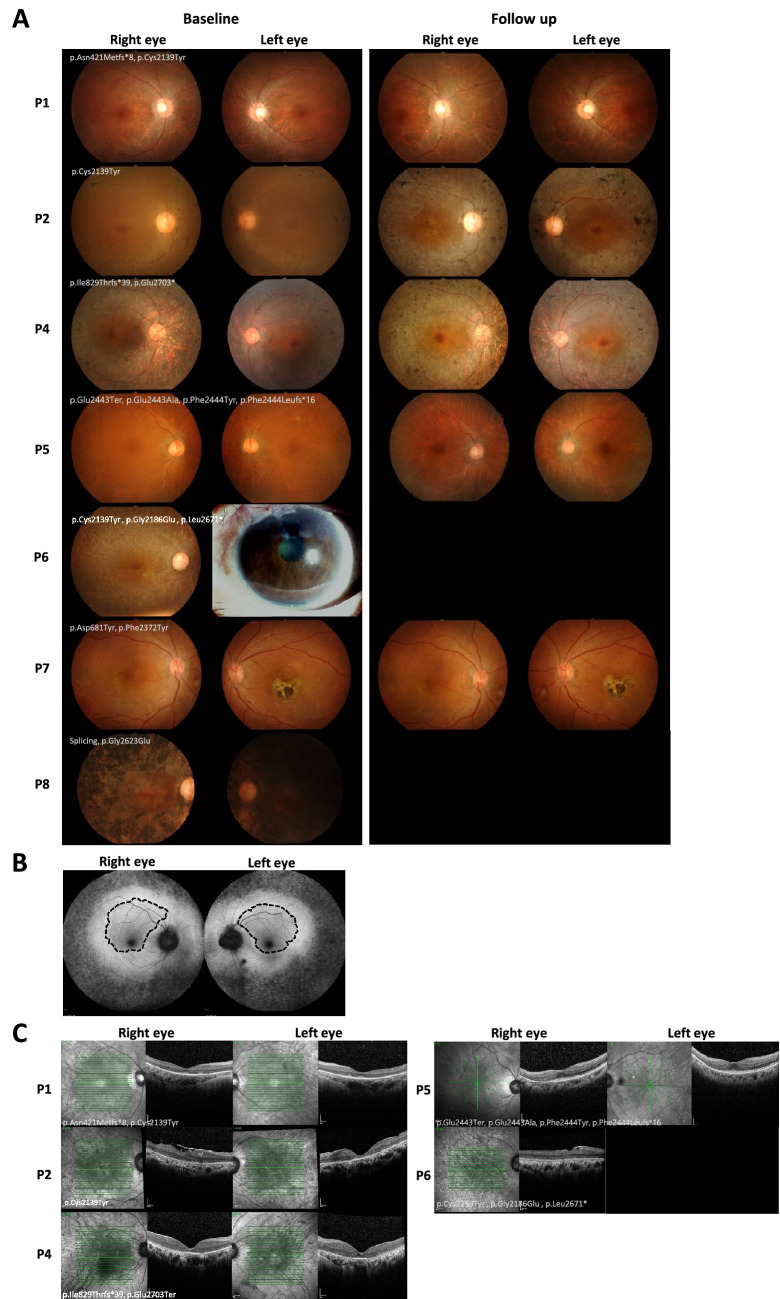
Fundus photography and optical coherence tomography (OCT) of patients with *EYS* gene variants**.** (**A**) Fundus photographs taken from patients at baseline and at last follow up displaying retinitis pigmentosa (RP) disease progression (except for P6 and P8). (**B**) Fundus autofluorescence of patient 5 showed a hyperautofluorescent ring along the outer border of the macula (dashed line), with a well-preserved central foveal structure. (**C**) OCT of five patients at follow up.

Patient 1, a 29-year-old female at the time of diagnosis, presented with reduced night vision in the past 10 years. Perimetry showed constricted VF, although her BCVA was well preserved. She had normal colour vision and a non-recordable ERG (Table 2). WES data revealed compound heterozygous mutations (p.Asn421Metfs*8 (Jinda, 2014), and p.Cys2139Tyr (VCV000189230.17)) in the *EYS* gene (Table 1, Supplementary Table S2), both of which were classified as pathogenic variants. At 10.7 years of follow up period, her BCVA remained relatively stable, but her VF was slightly reduced compared to baseline (Table 3). The fundus photography revealed a slight increase in hyperpigmentation along the retinal vessels and RPE atrophy (after 7.3 years of follow up time) compared to baseline (Fig. 3A). The OCT data (10.7 years follow up time) showed loss of ellipsoid zone except at the central fovea, thinning of outer nuclear layer, and attenuation of RPE in peripheral macular area. However, the inner retina was preserved and thickening of subfoveal Haller’s layer was noted.

**Table 3:** Follow up clinical manifestation.

Patient ID	Duration of follow up (years)	Eye	BCVA (logMAR)		VF at last follow up*	Intraocular pressure (mmHg)		IOL	Other ophthalmological condition	Treatment/intervention during follow up period
			Onset	Last visit		Onset	Last visit			
P1	10.7	OD	0.18	0.20	decreased	13	12	No	None	
		OS	0.18	0.20	decreased	14.5	10	No		
P2	9.2	OD	0.60	1.9	stable	14	9	Yes	Cataract NS + 1	Cataract removal & IOL
		OS	0.48	0.48	improved	14	12	Yes		
P3	0.08	OD	0.78	0.60	NE	16.5	16	No	Cataract mild NS	
		OS	0.60	0.78	NE	17	15	No		
P4	22	OD	0.60	2.7	NE	17	15	Yes	Cataract mild NS	Cataract removal & IOL
		OS	0.48	3	NE	17	15	Yes		
P5	11.7	OD	0.20	0.20	decreased	14	15	Yes	Cataract mild NS + PSC	Cataract removal & IOL
		OS	0.20	0.20	decreased	15	15	Yes		
P6	15.7	OD	2.3	2.3	NE	15	15	Yes	Absolute glaucoma	Trabeculectomy
		OS	2.7	2.7	NE	10	19	Yes		
P7	6.3	OD	0.60	0.50	NE	NE	17	No	None	
		OS	0.60	0.90	NE	NE	17	No		

*In comparison to baseline clinical data.

BCVA, best corrected visual acuity; IOL, intraocular lens; logMAR, logarithm of the minimum angle of resolution; NE, not evaluated; NS, nuclear sclerotic (cataract); OD, oculi dextra; OS, oculi sinistra; PSC, posterior subcapsular cataract; VF, visual field.

Patient 2 and 3, a female diagnosed at 49 and a male diagnosed at 50 years, respectively, presented with reduced night vision. Both had a moderate BCVA decrease and total colour blindness at baseline (Table 2). Mutation analysis revealed homozygous mutation in the *EYS* gene. Patient 2 had a homozygous pathogenic missense mutation at position p.Cys2139Tyr (VCV000189230.17), located in the EGF-like 21 (Fig. 2A, Table 1). Patient 2 showed conspicuous reduction of BCVA in the right eye and constricted VF both eyes throughout a follow up period of 9.2 years. Her fundus photography showed progressive RPE atrophy and increased hyperpigmentation compared to baseline (Fig. 3A). The OCT obtained at 9.2 years of follow up time revealed the development of epiretinal membrane on the retinal surface of the right eye resulting in distorted foveal contour. We also noticed the disappearance of ellipsoid zone in both eyes and the reduction in central foveal thickness of the left eye (Fig. 3C). Although patient 2 and 3 showed similar clinical presentations at the baseline, the genotypic presentation of patient 3 was a nonsense mutation at position p.Glu2703* (VCV000853127.6) which was classified as pathogenic variant (Table 1). Unfortunately, the fundus photography of patient 3 was not available and, with only one month follow up, the clinical data did not deviate from the baseline (Table 3).

Patient 4 presented with a feeling of narrowing VF at the age of 42. At baseline, she had a moderate BCVA decrease and total colour blindness in both eyes (Table 2). Mutation analysis revealed two pathogenic mutations in the *EYS*: one non-reported frameshift (pIle8297Thrfs*39) in the EGF-like 11 and one non-sense mutation at the same position as patient 3 (p.Glu2703*, VCV000853127.6). In addition to the *EYS* gene, a non-reported missense mutation in the *USH2A* gene was also detected (p.Gly774Glu) (Table 1, Supplementary Table S2). This variant was classified as VUS (based on the ACMG classification). As the disease progressed after 16 years of follow up, she lost the ellipsoid zone, the outer nuclear layer was thinned at central macula and absent at the periphery, and the outer plexiform layer was absent in the peripheral macula. After a follow up period of 22 years, she had a notable decline in the BCVA (Table 3). Her fundus photography showed extensive RPE atrophy, constricted retinal arterioles, scattered bone spicule pigmentation (Fig. 3A), and eventually a bull’s eye macula resulting from perifoveal hypopigmentation. She lost her light perception vision at the age of 72.

Patient 5 was diagnosed at the age of 50 years. This male patient presented with reduced night vision in addition to blurred vision and impaired depth perception. Perimetry revealed a considerable reduction in the visual field with well-preserved BCVA (logMAR of 0.2) (Table 2). We detected four non-reported mutations in the *EYS* gene in this patient. Two pathogenic variants (p. Glu2443* and p. Phe2444Leufs*16) and two VUS (p.Glu2443Ala, and p.Phe2444Tyr) (Table 1, Supplementary Table S2). Over the follow up of 11.7 years, this patient also showed reduction in the VF with stable BCVA (logMar of 0.2), both eyes (Table 3). The fundus photography (at 8.6 years of follow up) showed increased RPE atrophy compared to baseline (Fig. 3A). Fundus autofluorescence images showed a broad ring of hyperautofluorescence along the outer border of the macula with mottling hyper- and hypopigmented spots outside the macula in both eyes (Fig. 3B). The OCT revealed loss of ellipsoid zone and thinning of outer nuclear layer in the inferior macula (Fig. 3C).

Patient 6, diagnosed at the age of 46, presented with blurred vision since she was 15 years old. In addition, she complained of VF narrowing and reduced night vision. The BCVA at baseline was very poor (legally blind; BCVA LogMAR ≥ 1.00), thus making the VF and colour vision unable to be examined (Table 2). Fundus photography of patient 6 showed mottled hypopigmentation and markedly attenuated retinal vessels (Fig. 3A). The genotypic testing revealed that patient 6 had three reported mutations in the *EYS*; p.Cys2139Tyr (VCV000189230.17); p.Gly2186Glu (VCV000143108.4); p.Leu2671* (VCV000143113.3), all of which were classified as pathogenic variants. One mutation (p.Cys2139Tyr), located in EGF-like 21, was shared with patient 1 and patient 3. A non-reported likely-pathogenic variant in the *SNRNP200* gene (p.Arg110Gln), an autosomal dominant RP gene, was also observed in this patient (Supplementary Table S2). The OCT demonstrated an epiretinal membrane causing distorted contour of the foveal dimple. Loss of ellipsoid zone, thinning of outer nuclear layer, and faint outer plexiform layer were also shown (Fig. 3C).

Patient 7 presented with blurred vision at the age of 46 years. She had a moderate decrease in the BCVA and abnormal colour vision at baseline (Table 2). Three non-reported variants (in ClinVar) were detected in this patient, two of which were found in the EGF-like 8 domain (p.Asp681Tyr (novel)) and EGF-like 23 domain (p.Phe2372Tyr (rs1004559050)) of the *EYS* gene. Another novel mutation was detected in the *BEST1* gene (p.Ala10Pro) (Table 1, Supplementary Table S2). After a follow up of 6.3 years, she had a stable BCVA in the right eye and reduction in the left eye (Table 3). Her fundus photography showed mottled hypopigmentation in the right eye and chorioretinal scars in the fovea of the left eye, which remained stable over the course of follow up (Fig. 3A).

## Discussion

Although IRD affected populations worldwide, the prevalence of causative genes and their variants were different among ethnic groups. We previously reported a pilot study (N = 20) identifying the variant-associated IRDs, including RP, Leber congenital amaurosis (LCA), and cone-rod dystrophy (CRD) in Thai patients^14^. Here, we updated the prevalence of IRD-related genes with the larger number of patients (N = 125) with some variants that have never been listed in ClinVar database. In our patients, the variations in the *EYS* gene were identified as the highest gene contributing to this disease. Therefore, we further analyzed the genotypic and phenotypic profiles of 7 solved patients with *EYS* gene variants.

The *EYS* c.6416G>A (p.Cys2139Tyr), which is commonly found in both Caucasian and Asian population, is the most prevalent pathogenic variant found in our cohort^17–20^. This protein consists of 27 EGF-like domains, each consisting of six cysteine residues (Fig. 2A and Supplementary Fig. S1). Cysteine plays important role in maintaining protein conformation and stabilization by forming disulfide bonds with other cysteine residues, thus variations resulting in alteration of these residues often result in deleterious effects on the protein. One example is the nucleotide variation altering the last cysteine residue in the EGF-L21 domain into tyrosine. Originally, in the wild type EGF-L domain, Cys2139 residue forms a disulfide bond with Cys2130 (Supplementary Fig. S1). It is possible that by losing one of the cysteine residues, the unpaired cysteine forms a disulfide bridge with other cysteine residues, resulting in protein missfolding and impairment of protein function. A 3D structure modeling on Cys2139Tyr also described that the amino acid variation altered the protein conformation and affected its solubility^21^. In our cohort, this variant was the most prevalent and suspected to be the cause of the disease in 3 patients (Patient 1, 2 and 6). One of the patients, patient 2, carried a homozygous Cys2139Tyr. Even though showing a late onset, the disease manifested as a fast progression as the blurred vision and total colour blindness developed within 3 years after onset, accompanied by the disappearance of the ellipsoid zone (EZ) in both eyes (Fig. 3C) after 9 years of follow up. Accordingly, the fast disease progression was also reported in a study of a Chinese family harbouring the same variant^21^.

Patient 6, manifesting with an early disease onset, harboured triple pathogenic variants in the *EYS* gene, two missense (p.Cys2139Tyr and p.Gly2186Glu) and one nonsense (p.Leu2671*) variant, and a VUS in *SNRNP200* (p.Arg110Gln). The same two missense variants in the *EYS* were also reported in an affected Chinese family in a previous study^20^, although the affected individuals did not show early onset manifestations as seen in our patient. Thus, the early onset feature could be attributed to the nonsense variant (p.Leu2671*) of the *EYS*, the different genetic background, or the variant in *SNRNP200*, a gene causing autosomal dominant RP. The latter had been reported to manifest at an early age^22^. However, the segregation analysis in our patient did not indicate the evidence of vertical inheritance and the variant is not a null or occur in an important domain. Thus, it is unlikely that the *SNRNP200* variant contributed to the disease phenotype in this case.

Three of our patients, patient 3, 4, and 5, presented with multiple nonsense variants in both alleles resulting in null variant, although we observed some different features in their phenotypes. The deletion of one or more of the highly conserved Laminin G-like and EGF-like domains had been reported to be associated with the high disease prevalence^11,23,24^. In our cohort, the homozygous null variant, p.Glu2703*, was identified in patient 3. The variant produced a defective EYS protein with deletions of two Laminin G-like and two EGF-like domains in the C-terminus (Fig. 2A). Incongruously, compound heterozygous variants, p.Glu2703* and p.Ile829Thrfs*39 were identified in patient 4. The later variant produces a very shortened protein with only 26% of total protein length preserved in the N-terminus. This variant lost all the Laminin G-like domains and more than half of the EGF-like domains which were replaced by 39 frameshifted residues. Phenotypically, patient 4 lost the ellipsiod zone after 16 years and the light perception after 20 years follow up, respectively.

In patient 5, quadruple variants were detected at two locations: 2443 and 2444. This resulted in 2 possible combinations where either the null variants reside on the same or different allele. Both seem to be equally deleterious as they occurred at only one residue away from each other. The fundus autofluorescence (FAF) of patient 5 showed a hyperautofluorescent ring along the outer border of the macula, with a well-preserved central foveal structure (Fig. 3B) even after 7 years of follow up, resulting in stable BCVA. This finding corresponded with the report of a distinct phenotype of crescent ring on the FAF in patients harbouring variants clustering at the C-terminus of the EYS. The shape of the hyperautofluorescent ring (Fig. 3B, circled with dash lines in both eyes) was hypothesized to reflect areas of the preserved retinal thickness and ellipsoid zone^24^.

The phenotypic variation between the null *EYS* variants could be explained by the nonsense-mediated mRNA decay (NMD), an mRNA quality control function that neglects the expression of defected genes, which was reported to occur in a varying degree depending on the *EYS* mutation. Some truncated variants can partially or majorly escape the NMD. The escaped mRNA, in turn, produces a truncated EYS protein that still provides some function^25^.

Patient 8 harboured one likely pathogenic splicing variant, IVS19+1G>A, and a VUS, p.Gly2623Gl, in *EYS* gene (Supplement Table S2). In addition to the variant in *EYS*, we identified three VUS variants (p.Ser555Asn (RIMS1), Val469Phe (CNGA1), Glu1154Val (IMPG2) in this patient (Supplement Table S2). We observed extensive retinal hyperpigmentation encroaching the foveal centre in both eyes (Fig. 3A) which corresponded to a study that reported heavy pigmentation in the macular area in patients with two *EYS* and one *RIMS1* variant^26^. However, according to the classification of the clinical significance (see methods), we did not confirm that the variants in the *EYS* gene were the cause of disease in this patient. Future evidence from in vitro or in vivo experiments, along with more reports of phenotypic findings in patients, could be used to re-evaluate and update the clinical classification of these VUS.

The AF of half of the *EYS* variants found in this study had been reported in gnomAD—Exomes. All variants showed a higher AF in the Asian compared to the global population with one exception for a likely benign variant, c.904C>T. Moreover, two variants, c.6557G>A and c.3293C>A, were considered extremely rare (minor allele frequency, MAF < 0.01%) in the global population (0.004% and 0.0045%, respectively) compared to the Asian population (0.019% and 0.018%, respectively). This data suggested ethnical specificity of the variants. Some Asian-specific variants were also observed in a study of the Japanese population^27^. The combined results brought forth the hypothesis that *EYS* plays a crucial role in IRD in the Asian population. Thus, it poses as a promising therapeutic target, particularly for a larger Asian population, though more data from other settings beside Thailand and Japan are mandatory needed.

Beyond the study of genotype–phenotype correlation, gene rescue therapy options for various IRD-related genes were being developed during the past decade^28^. At present, the adeno-associated viral (AAV) vector is one of the most studied gene therapy platforms. Unfortunately, a large gene such as *EYS* spans with a coding size of more than twice of the packing capacity of the AAV vectors, making it difficult to utilise this technique. As such, the options were narrowed down to the genome editing technology, including the use of the clustered regularly interspaced short palindromic repeats (CRISPR) system that had been attempted in the zebrafish model^29^. The induced pluripotent stem cell (iPSC) replacement therapy could also be considered albeit some challenges on cell differentiation and neural connection^30^. Thus, our findings on the *EYS* variant profile in the Thai population could serve as a foundation for further research on these novel techniques. In the future, we continue to strive in exploring the IRD-related genes using the whole genome sequencing to update the gene panels to expand the knowledge and data on IRD patients. Lastly, although our study found that patients with *EYS* variations showed the late onset of disease, the family members at risk are recommended to seek the counselling and undergo the ophthalmological check-up regularly at least once a year. They might also consider genetic testing to identify the variations of *EYS* and other IRD-related genes.

## Supplementary Information

Supplementary Figure S1.

Supplementary Tables.

## Data Availability

The datasets generated and/or analysed during the current study are available in ClinVar (https://www.ncbi.nlm.nih.gov/clinvar/?term=SUB12291838[Submitter+Batch]). The accession numbers for the ClinVar submission are SCV002754564 to SCV002754576.
