# Supplementary material for: Genotypic and phenotypic profiles of EYS gene-related retinitis pigmentosa: a retrospective study
Source: Sci Rep. 2022 Dec 13;12:21494. doi: 10.1038/s41598-022-26017-0 (PMC9748023; doi:10.1038/s41598-022-26017-0)
Supplement: Supplementary file 1 — Supplementary Figure S1. [file 41598_2022_26017_MOESM1_ESM.pdf]

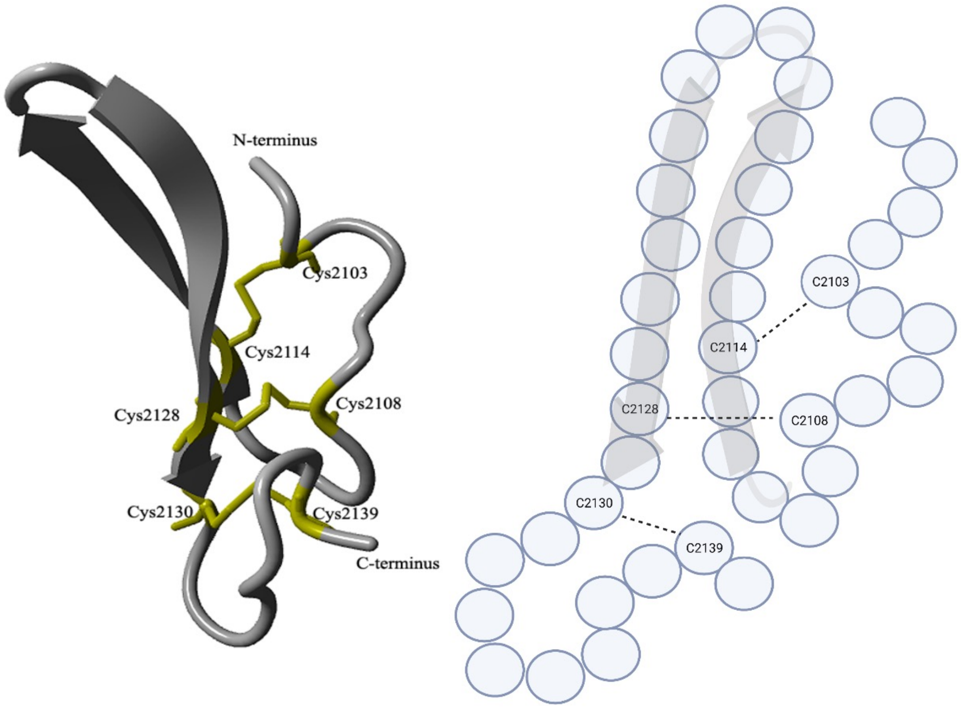

Supplementary Figure S1. Schematic representation of the predicted structure of EGF-L21 domain of the EYS gene. The EGF-L21 domain consists of six cysteine residues, which forms paired disulfide bonds under normal conditions. Mutations altering a cysteine residue into another amino acid might result in unpaired cysteine, thus forcing it to bind other residues, resulting in protein misfolding. The 3D structure of the EGF-L21 (left) was predicted by swissmodel.expasy.com and rendered using YASARA software. The EGF-L21 illustration (right) was created using Biorender.
