# Supplementary material for: Genotypic and phenotypic profiles of EYS gene-related retinitis pigmentosa: a retrospective study
Source: Sci Rep. 2022 Dec 13;12:21494. doi: 10.1038/s41598-022-26017-0 (PMC9748023; doi:10.1038/s41598-022-26017-0)
Supplement: Supplementary file 2 — Supplementary Tables. [file 41598_2022_26017_MOESM2_ESM.docx]

**Supplementary Table S1. List of identified IRD-associated genes in RP patients**

| **No** | **Gene** | **Number of variants** | **Percentage** | **No** | **Gene** | **Number of variants** | **Percentage** |
| --- | --- | --- | --- | --- | --- | --- | --- |
| 1 | *EYS** | 22 | 8.5 | 58 | *ADAM9* | 1 | 0.4 |
| 2 | *USH2A** | 22 | 8.5 | 59 | *C21orf2** | 1 | 0.4 |
| 3 | *ABCA4** | 15 | 5.8 | 60 | *C8orf37** | 1 | 0.4 |
| 4 | *CRB1** | 10 | 3.9 | 61 | *CABP4* | 1 | 0.4 |
| 5 | *CEP290** | 8 | 3.1 | 62 | *CLN3* | 1 | 0.4 |
| 6 | *RP1** | 6 | 2.3 | 63 | *CLRN1* | 1 | 0.4 |
| 7 | *GPR179* | 5 | 1.9 | 64 | *CLUAP1* | 1 | 0.4 |
| 8 | *GUCY2D* | 5 | 1.9 | 65 | *CNGB3* | 1 | 0.4 |
| 9 | *BEST1** | 4 | 1.6 | 66 | *CNNM4* | 1 | 0.4 |
| 10 | *C2orf71** | 4 | 1.6 | 67 | *ELOV4* | 1 | 0.4 |
| 11 | *CNGB1* | 4 | 1.6 | 68 | *GPR125* | 1 | 0.4 |
| 12 | *FAM161A** | 4 | 1.6 | 69 | *GUCA1B** | 1 | 0.4 |
| 13 | *HMCN1** | 4 | 1.6 | 70 | *KIAA0556** | 1 | 0.4 |
| 14 | *KIAA1549* | 4 | 1.6 | 71 | *KLHL7** | 1 | 0.4 |
| 15 | *PNPLA6** | 4 | 1.6 | 72 | *LCA5* | 1 | 0.4 |
| 16 | *RBP3* | 4 | 1.6 | 73 | *MYO7A* | 1 | 0.4 |
| 17 | *RP1L1** | 4 | 1.6 | 74 | *NR2E3* | 1 | 0.4 |
| 18 | *SPATA7** | 4 | 1.6 | 75 | *OFD1** | 1 | 0.4 |
| 19 | *TRPM1* | 4 | 1.6 | 76 | *PAX6** | 1 | 0.4 |
| 20 | *ALMS1** | 3 | 1.2 | 77 | *PDE6A** | 1 | 0.4 |
| 21 | *CDHR1* | 3 | 1.2 | 78 | *PRDM13** | 1 | 0.4 |
| 22 | *CYP4V2* | 3 | 1.2 | 79 | *PRPF31** | 1 | 0.4 |
| 23 | *EMC1** | 3 | 1.2 | 80 | *PRPF4** | 1 | 0.4 |
| 24 | *MAK* | 3 | 1.2 | 81 | *PRPF8** | 1 | 0.4 |
| 25 | *PDE6B* | 3 | 1.2 | 82 | *RAB28* | 1 | 0.4 |
| 26 | *PITPNM1** | 3 | 1.2 | 83 | *RABL5* | 1 | 0.4 |
| 27 | *PROM1** | 3 | 1.2 | 84 | *RGR* | 1 | 0.4 |
| 28 | *RIMS1** | 3 | 1.2 | 85 | *RLBP1* | 1 | 0.4 |
| 29 | *SNRNP200** | 3 | 1.2 | 86 | *RPE65* | 1 | 0.4 |
| 30 | *UNC45B** | 3 | 1.2 | 87 | *RPGRIP1** | 1 | 0.4 |
| 31 | *AKAP9** | 2 | 0.8 | 88 | *SAG* | 1 | 0.4 |
| 32 | *CACNA2D4* | 2 | 0.8 | 89 | *SIX6** | 1 | 0.4 |
| 33 | *CERKL* | 2 | 0.8 | 90 | *TTC8* | 1 | 0.4 |
| 34 | *CHM** | 2 | 0.8 | 91 | *ZNF513* | 1 | 0.4 |
| 35 | *CNGA1* | 2 | 0.8 |  |  |  |  |
| 36 | *CRX** | 2 | 0.8 |  |  |  |  |
| 37 | *CTNNA1* | 2 | 0.8 |  |  |  |  |
| 38 | *FSCN2* | 2 | 0.8 |  |  |  |  |
| 39 | *GRK1* | 2 | 0.8 |  |  |  |  |
| 40 | *GRM6* | 2 | 0.8 |  |  |  |  |
| 41 | *GUCA1A** | 2 | 0.8 |  |  |  |  |
| 42 | *IFT172* | 2 | 0.8 |  |  |  |  |
| 43 | *IMPG2* | 2 | 0.8 |  |  |  |  |
| 44 | *IQCB1** | 2 | 0.8 |  |  |  |  |
| 45 | *KCNV2* | 2 | 0.8 |  |  |  |  |
| 46 | *MERTK** | 2 | 0.8 |  |  |  |  |
| 47 | *NCL** | 2 | 0.8 |  |  |  |  |
| 48 | *NMNAT1** | 2 | 0.8 |  |  |  |  |
| 49 | *OAT** | 2 | 0.8 |  |  |  |  |
| 50 | *PDE6C** | 2 | 0.8 |  |  |  |  |
| 51 | *RD3* | 2 | 0.8 |  |  |  |  |
| 52 | *RHO** | 2 | 0.8 |  |  |  |  |
| 53 | *RP2** | 2 | 0.8 |  |  |  |  |
| 54 | *TULP1* | 2 | 0.8 |  |  |  |  |
| 55 | *VCAN** | 2 | 0.8 |  |  |  |  |
| 56 | *WDR19** | 2 | 0.8 |  |  |  |  |
| 57 | *ZEB1** | 2 | 0.8 |  |  |  |  |
| * Potential causative genes | | | | | | | |

**Supplementary Table S2. EYS variants identified in RP patients**

| **Patient** | **Gene** | **Nucleotide change** | **Amino acid change** | **Zygosity** | **MOI** | **Variant classification** |
| --- | --- | --- | --- | --- | --- | --- |
| **Patients with EYS-causative bi-allelic variants** | | | | | | |
| P1 | *EYS* | c.1260_1260delG | p.Asn421Metfs*8 | Comp Het | arRP | P |
|  | *EYS* | c.6416G>A | p.Cys2139Tyr | Comp Het | arRP | P |
| P2 | *EYS* | c.6416G>A | p.Cys2139Tyr | Hom | arRP | P |
| P3 | *EYS* | c.8107G>T | p.Glu2703* | Hom | arRP | P |
| P4 | *EYS* | c.2486_2486delT | p.Ile829Thrfs*39 | Comp Het | arRP | P |
|  | *EYS* | c.8107G>T | p.Glu2703* | Comp Het | arRP | P |
|  | *USH2A* | c.2321G>A | p.Gly774Glu | Het | arRP, arUSH | VUS |
| P5 | *EYS* | c.7327G>T | p.Glu2443* | Comp Het | arRP | P |
|  | *EYS* | c.7328A>C | p.Glu2443Ala | Comp Het | arRP | LP |
|  | *EYS* | c.7331T>A | p.Phe2444Tyr | Comp Het | arRP | LP |
|  | *EYS* | c.7332_7333delCC | p.Phe2444Leufs*16 | Comp Het | arRP | P |
| P6 | *EYS* | c.6416G>A | p.Cys2139Tyr | Comp Het | arRP | P |
|  | *EYS* | c.6557G>A | p.Gly2186Glu | Comp Het | arRP | P |
|  | *EYS* | c.8012T>A | p.Leu2671* | Comp Het | arRP | P |
|  | *SNRNP200* | c.329G>A | p.Arg110Gln | Het | adRP | VUS |
| P7 | *EYS* | c.2041G>T | p.Asp681Tyr | Comp Het | arRP | LP |
|  | *EYS* | c.7115T>A | p.Phe2372Tyr | Comp Het | arRP | LP |
|  | *BEST1* | c.28G>C | p.Ala10Pro | Het | adMD | VUS |
| **Patients with non-disease causing EYS variants** | | | | | | |
| P8 | *EYS* | IVS19+1G>A | splicing | Comp Het | arRP | LP |
|  | *EYS* | c.7868G>A | p.Gly2623Glu | Comp Het | arRP | VUS |
|  | *RIMS1* | c.1664G>A | p.Ser555Asn | Het | adCRD | VUS |
|  | *CNGA1* | c.1405G>T | p.Val469Phe | Het | arRP | VUS |
|  | *IMPG2* | c.3461A>T | p.Glu1154Val | Het | arRP | VUS |
| P9 | *EYS* | c.904C>T | p.Leu302Phe | Comp Het | arRP | LB |
|  | *EYS* | c.3293C>A | p.Ala1098Glu | Comp Het | arRP | VUS |
|  | *CNGB3* | c.2020A>G | p.Lys674Glu | Het | arCRD | VUS |
| P10 | *EYS* | c.8107G>T | p.Glu2703Ter | Het | arRP | P |
|  | *KIAA1549* | c.4217G>A | p.Arg1406His | Het | arRP | VUS |
| P11 | *ELOV4* | c.617C>A | p.Pro206Gln | Het | adStargardt | VUS |
|  | *RIMS1* | c.2274G>C | p.Gln758His | Het | adCRD | LP |
|  | *IMPG2* | c.2985C>G | p.Asn995Lys | Het | arRP | VUS |
|  | *EYS* | c.3473G>A | p.Cys1158Tyr | Het | arRP | VUS |
| P12 | *CDHR1* | c.571C>T | p.Arg191Cys | Hom | arCRD | LP |
|  | *EYS* | c.9197T>C | p.Leu3066Pro | Het | arRP | VUS |
| P13 | *EYS* | c.904C>T | p.Leu302Phe | Comp Het | arRP | LB |
|  | *EYS* | c.3293C>A | p.Ala1098Glu | Comp Het | arRP | VUS |
|  | *CNGB3* | c.2020A>G | p.Lys674Glu | Het | arOther retinopathy | VUS |
| P14 | *IFT172* | c.406C>T | p.Arg136Cys | Comp Het | arBardet-Biedl syndrome, arRP | VUS |
|  | *IFT172* | c.1535A>G | p.Tyr512Cys | Comp Het | arBardet-Biedl syndrome, arRP | LP |
|  | *EYS* | c.6416G>A | p.Cys2139Tyr | Het | arRP | P |
| P15 | *HMCN1* | c.7079G>A | p.Arg2360His | Het | adMD, AMD | LB |
|  | *EYS* | c.8780_8783delGTAT | p.Cys2927Tyrfs*47 | Het | arRP | LP |
| P16 | *USH2A* | c.4732C>T | p.Arg1578Cys | Comp Het | arRP, arUSH | LP |
|  | *USH2A* | c.13649T>G | p.Val4550Gly | Comp Het | arRP, arUSH | VUS |
|  | *RHO* | c.586C>A | p.Pro196Thr | Het | adRP, arRP, adCSNB | VUS |
|  | *EYS* | c.7868G>A | p.Gly2623Glu | Het | arRP | VUS |
| P17 | *PITPNM1* | c.161G>A | p.Gly54Asp | Het | adRD | LP |
|  | *EYS* | c.7065A>C | p.Glu2355Asp | Het | arRP | VUS |
|  | *CLRN1* | c.407G>A | p.Gly136Glu | Het | arRP, arUSH | VUS |
|  | *CLUAP1* | c.496C>T | p.Arg166Trp | Het | arLCA | VUS |
| P18 | *EYS* | IVS19+1G>A (c.2993+1G>A) | splice donor site change | Comp Het | arRP | LP |
|  | *EYS* | c.7868G>A | p.Gly2623Glu | Comp Het | arRP | VUS |
|  | *RIMS1* | c.1664G>A | p.Ser555Asn | Het | adCRD | VUS |
|  | *CNGA1* | c.1405G>T | p.Val469Phe | Het | arRP | VUS |
|  | *IMPG2* | c.3461A>T | p.Glu1154Val | Het | arRP | VUS |
